# Long-Term Immunogenicity of Rabies Pre-Exposure Prophylaxis in Japanese Adult Travelers: Comparison of Dosing Regimens †

**DOI:** 10.3390/vaccines13111169

**Published:** 2025-11-17

**Authors:** Shinji Fukushima, Akira Nishizono, Takehiro Hashimoto, Atsuo Hamada

**Affiliations:** 1 Travellers’ Medical Center, Tokyo Medical University Hospital, 6-7-1 Nishi-shinjuku, Shinjuku-ku, Tokyo 160-0023, Japan; a-hamada@tokyo-med.ac.jp; 2 Department of Microbiology, Faculty of Medicine, Oita University, 1-1 Idaigaoka, Hasama-machi, Yufu, Oita 879-5593, Japan; a24zono@oita-u.ac.jp (A.N.); hashimo2013@oita-u.ac.jp (T.H.); 3 Research Center for Global and Local Infectious Diseases, Oita University Hospital, 1-1 Idaigaoka, Hasama-machi, Yufu, Oita 879-5593, Japan; 4 Hospital Infection Control Center, Oita University Hospital, 1-1 Idaigaoka, Hasama-machi, Yufu, Oita 879-5593, Japan

**Keywords:** rabies, rabies vaccine, pre-exposure prophylaxis, long-term immunogenicity, anti-rabies antibody level

## Abstract

Background/Objectives: Japan is a rabies-free country; therefore, pre-exposure prophylaxis (PrEP) is primarily recommended for travelers to rabies-endemic regions. However, no prior studies in Japan have assessed long-term immunogenicity after PrEP vaccination. Methods: This descriptive study evaluated the long-term persistence of rabies virus-neutralizing antibodies among Japanese adult travelers who had received PrEP. Neutralizing antibody levels were measured using the rabies rapid fluorescent focus inhibition test more than two years post-vaccination. Results: Among 97 participants, 86.6% remained seropositive, with a median interval of 4 years since the last vaccination. Individuals who received three or more doses had significantly higher geometric mean titers than those who received only two doses. A notable proportion of those vaccinated with PCECV-KMB, an older subcutaneous formulation, were seronegative after a long interval. Conclusion: Antibody levels were strongly influenced by the number of vaccine doses, with reduced persistence in those who received only two. If testing confirms sufficient titers, PrEP booster doses may not be needed. However, for individuals with only two doses, older vaccinations, or those given PCECV-KMB, a risk-based assessment is recommended—especially for travelers to rabies-endemic areas.

## 1. Introduction

Rabies is a zoonotic disease caused by lyssaviruses of the family *Rhabdoviridae* and is primarily transmitted to humans through the bites of infected animals, particularly dogs [1,2,3]. It remains a significant global public health concern, with an estimated 59,000 human deaths occurring annually—most of which are reported in Africa and Asia [4]. Although the burden is highest in these regions, sporadic cases are also documented in the Americas and Europe. For instance, the United States reports approximately 1 to 3 human rabies deaths per year, primarily associated with bat exposure. In contrast, Europe maintains a low incidence of rabies through comprehensive surveillance and widespread vaccination programs.

Once clinical symptoms appear, rabies is almost universally fatal, with a case fatality rate approaching 100%. The disease typically progresses through several stages: an incubation period lasting weeks to months, followed by a prodromal phase with flu-like symptoms, and then an acute neurologic phase characterized by agitation, hallucinations, hydrophobia, and paralysis. Death usually occurs within days to weeks after symptom onset due to respiratory failure.

However, the occurrence of rabies in humans and animals can be prevented by vaccination [5]. The main rabies vaccines used in many countries are cell culture vaccines, such as purified Vero cell rabies vaccine (PVRV), human diploid cell rabies vaccine, and purified chick embryo cell rabies vaccine (PCECV). The World Health Organization (WHO) recommends pre-exposure prophylaxis (PrEP) and post-exposure prophylaxis (PEP) vaccination regimens for the prevention of rabies in humans [6,7]. A previous PrEP regimen included three doses (0, 7, 21–28 days), and PEP consists of five doses [8]. In many countries, modern PrEP consists of two doses and PEP consists of four doses [6].

Japan is a rabies-free island nation, with no domestic cases reported since the 1950s, except for imported human rabies cases [9,10]. Consequently, individuals residing in Japan do not receive rabies vaccination for either PrEP or PEP. Rabies vaccination among Japanese individuals is limited to those who undergo PrEP prior to traveling to rabies-endemic countries or those who receive PEP following animal bites in such regions. In Japan, travel clinics not only administer vaccines that are officially approved for domestic use, but also individually import vaccines from overseas to accommodate the immunization recommendation of international travelers. This practice enables the provision of vaccines that are recommended or mandated by destination countries but have not yet been licensed in Japan. For example, in the context of rabies pre-exposure prophylaxis, several internationally recognized vaccines—including Verorab, Chirorab, Abhayrab, and SPEEDA—are commonly imported and administered to travelers requiring protection against rabies in endemic regions.

Historically, the human rabies vaccine PCECV-KMB was used in Japan; however, its distribution has been discontinued. In 2019, Rabipur was approved and has since become the standard rabies vaccine in Japan. Notably, rabies immune globulin is not approved for use within Japan. Furthermore, rabies virus neutralizing antibody titers cannot be measured outside of specialized research institutions in Japan.

Under these circumstances, rabies PrEP plays a critical role in protecting Japanese travelers from rabies. Nevertheless, data on the long-term persistence of anti-rabies antibodies following PrEP among Japanese travelers remain limited. This study aimed to evaluate the long-term immunogenicity of rabies PrEP vaccination in Japanese adult travelers.

## 2. Materials and Methods

### 2.1. Study Design and Trial Registration

This descriptive study was conducted from 27 July 2020 to 31 March 2023 at Tokyo Medical University Hospital in Japan. The study was registered with the Clinical Trial Registry (UMIN000040525) on 26 May 2020 before Japanese adult participants were registered for the study.

### 2.2. Participants

Eligible participants were adults aged 20 years or older who had received rabies PrEP more than two years ago. Vaccinations administered in Japan or abroad were included, regardless of the number of doses received. Individuals under 20 years of age at enrollment or with a history of rabies PEP were excluded. All participants were recruited from the Travellers’ Medical Center at Tokyo Medical University Hospital, where individuals voluntarily seek pre-travel health consultations.

### 2.3. Study Procedures

All participants signed an informed consent form before participating in the study. They received blood sampling after their eligibility was confirmed. They completed a questionnaire that included information about their sex, date of birth, and rabies PrEP vaccination history. Their rabies vaccination history was obtained from immunization records. The serum samples were stored at ≤−20 °C. All blood samples were shipped to Oita University (Oita, Japan) for the measurement of rabies virus neutralization antibodies.

### 2.4. Measurement of Rabies Virus Neutralization Antibodies

Neutralizing antibody levels against rabies virus were determined using the rabies rapid fluorescent focus inhibition test [8,11,12,13]. Each serum sample was diluted two-fold with Minimum Essential Medium that contained 2% fetal calf serum, and then placed in 1 well of a 96-well plate (Nunc, Roskilde, Denmark). The samples were set up in duplicate. CVS-11 (50% tissue culture infectious dose 100) was added to each well and incubated in a 5% CO_2_ incubator at 37 °C for 90 min. Next, BHK-21 cells were added to each well and incubated for 24 h. Finally, cells were fixed with 90% acetone, stained with FITC-conjugated anti-rabies N monoclonal antibody (Fuji Rebio Diagnostics, Inc., Malvern, PA, USA) at 37 °C for 45 min, and observed under a fluorescence microscope. The average values of duplicate samples were determined. The neutralizing antibody level was calculated by comparison with WHO international standards (RAI: anti-rabies immunoglobulin, human, National Institute for Biological Standards & Control). A neutralizing antibody level ≥0.5 IU/mL was defined as adequate for protection against rabies (WHO, 1992) [7].

### 2.5. Statistical Analysis

To evaluate the long-term immunogenicity of rabies vaccination, seroprotection rates, geometric mean titers (GMTs), and corresponding 95% confidence intervals (CIs) were calculated. Seroprotection was defined as a neutralizing antibody titer ≥0.50 IU/mL, in accordance with WHO guidelines. Antibody prevalence and GMTs were compared across subgroups stratified by sex, age at primary vaccination, and number of PrEP doses.

Fisher’s exact test was used for comparisons of antibody prevalence between two groups, while the Holm and Cochran–Armitage tests were applied for comparisons among multiple groups. GMTs were compared using the Wilcoxon rank-sum test for two-group comparisons and the Kruskal–Wallis test for comparisons across multiple groups. A two-sided *p*-value < 0.05 was considered statistically significant.

To evaluate the effects of multiple categorical factors on log-transformed antibody titers (LogAb), multi-way analysis of variance (ANOVA) was conducted using a linear model specified as lm(LogVNA~Factor1.AgeGroup + Factor2.VaccinationDoseGroup + Factor3.IntervalGroup + Factor4.Sex + Factor5.VaccineType). Type III sums of squares were applied to assess the unique contribution of each factor while controlling for the influence of the others. This approach is appropriate for unbalanced designs and allows for the evaluation of main effects independently of potential interactions.

All statistical analyses were conducted using EZR (version 1.68; Jichi Medical University, Tochigi, Japan), a graphical user interface for R (version 4.3.1) that incorporates commonly used biostatistical functions via R Commander (version 2.9-1) [14].

### 2.6. Ethical Considerations

This study was conducted in compliance with the principles of the Declaration of Helsinki and approved by the Institutional Review Board of the Tokyo Medical University (SH3607) on 28 February 2017. All participants provided written informed consent before enrollment.

## 3. Results

### 3.1. Characteristics of the Study Participants

The 97 participants comprised 53 men and 44 women, with a median age at primary vaccination of 35 years (interquartile range [IQR] 29–44 years), and median age at enrollment of 41 years (IQR 35–52 years). A total of 10 participants had received two doses of rabies vaccine, and 87 participants had received three or more doses of vaccine. Classification was based on the vaccine product administered for the first dose of the vaccination series. Specifically, 45 participants received the purified chick embryo cell vaccine (PCECV), and 51 participants received the purified Vero cell rabies vaccine (PVRV). The median time between the last dose of rabies vaccination and enrollment was 4 years (IQR of 3–7 years, range of 2–18 years) (Table 1).

### 3.2. Seroprotection Rate and GMT (Table 2)

As shown in Table 2, 84 of 97 participants were antibody-positive (86.6%). Of these, 24 (28.6%), 33 (39.3%), 9 (10.7%), and 18 (21.4%) had antibody levels of 0.5–0.99, 1.0–2.49, 2.5–3.99, and ≥4.0 IU/mL, respectively.

The proportion of participants who tested positive for antibodies was higher in participants who received three or more doses of vaccine (60.0% for those who received two doses, 86.2% for three doses, 94.1% for four doses, and 100% for five or more doses). This trend was statistically significant (*p* < 0.05).

The GMT was higher in participants who received more than three doses of vaccine (0.64 IU/mL for those receiving two doses, 1.15 IU/mL for three doses, 5.58 IU/mL for four doses, and 3.64 IU/mL for five or more doses), and this trend was statistically significant (*p* < 0.05).

**Table 2:** Seroprotection rate and geometric mean antibody level (GMT).

Characteristics	Seroprotection		GMT (95%CI)	
	n/N	% (95%CI)		
Total	84/97	86.6 (78.2–92.7)	1.65	(1.22–2.23)
Sex				
Male	46/53	86.8 (74.7–94.5)	1.81	(1.10–2.98)
Female	38/44	86.4 (72.6–94.8)	1.47	(1.09–1.99)
	*p* = 1.000		*p* = 0.951	
Age at primary vaccination (years)				
<50	75/85	88.2 (79.4–94.2)	1.67	(1.24–2.25)
≥50	9/12	75.0 (42.8–94.5)	1.52	(0.39–5.92)
	*p* = 0.201		*p* = 0.558	
Vaccination doses				
2	6/10	60.0 (26.2–87.8)	0.64	(0.31–1.33)
3	50/58	86.2 (74.6–93.9)	1.15	(0.88–1.51)
4	16/17	94.1 (71.3–99.9)	5.58	(1.85–16.8)
5 or more	12/12	100 (73.5–100)	3.64	(1.60–8.28)
	*p* < 0.05		*p* < 0.05	
Vaccine type				
PCECV-KMB	21/33	63.6 (45.1–79.6)	0.93	(0.57–1.51)
Rabipur	12/12	100 (73.5–100)	3.96	(0.92–17.1)
PVRV	50/51	98.0 (89.6–100)	1.95	(1.40–2.72)
Unknown	1/1		1.75	
	*p* < 0.05		*p* = 0.159	
Elapsed time from last vaccination (years)				
2–3	32/36	88.9 (73.9–96.9)	2.23	(1.25–3.97)
4–9	45/53	84.9 (72.4–93.3)	1.30	(0.90–1.88)
10–18	7/8	87.5 (47.3–99.7)	2.05	(0.78–5.41)
	*p* = 0.900		*p* = 0.444	

Fisher’s exact test was used to compare antibody prevalence rates between two groups, and the Holm test and Cochran–Armitage test were used to compare antibody prevalence rates between multiple groups. Comparisons of GMT between two groups were performed using the Wilcoxon rank sum test, and multi-group comparisons of GMT were performed using the Kruskal–Wallis test. Vaccine type: classification was based on the product used for the first dose of the vaccination series.

ANOVA was conducted to evaluate the effects of age group, dose group, interval group, sex, and vaccine type on log-transformed antibody titers (Log VNA). The analysis revealed that the number of doses administered (Factor2.VaccinationDoseGroup) had a statistically significant effect on LogAb levels (F(3, 86) = 10.26, *p* < 0.001). Vaccine type (Factor5.VaccineType) also showed a significant association with antibody titers (F(3, 86) = 4.27, *p* = 0.007). Elapsed time from last vaccination (Factor3.IntervalGroup) demonstrated a marginal effect (F(2, 86) = 2.84, *p* = 0.064), suggesting a potential trend. In contrast, age group (Factor1.AgeGroup) and sex (Factor4.Sex) did not show significant associations with LogVNA (*p* > 0.05) (Table 3).

### 3.3. The Relationship Between the Elapsed Time and Antibody Levels

**Figure 1:**
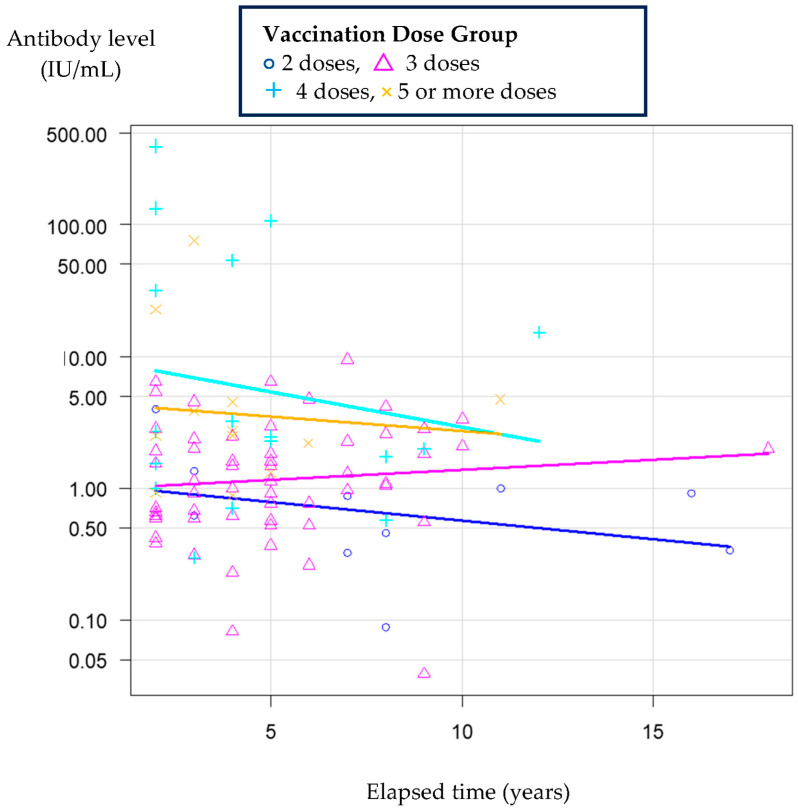
Relationship between the elapsed time and antibody levels by vaccine dose group. Scatter plots illustrate antibody titers over time since the last vaccination in each dose group. Colored lines indicate least squares regression lines fitted to each group: blue for the 2‑dose group, pink for the 3‑dose group, cyan for the 4‑dose group, and yellow for the 5‑or‑more‑dose group.

The relationship between the elapsed time (years) and antibody levels (IU/mL) by vaccine dose group is shown in Figure 1.

### 3.4. Anti-Rabies VNA Levels After Each Dose of Vaccine

#### 3.4.1. Two-Dose Sample

Among the 10 participants who received two doses of the rabies vaccine, 6 participants were antibody-positive (60%). Table 4 shows the characteristics of the participants who received two doses of vaccine, which included three who received PVRV and seven who received PCECV. The seroprotection rate declined as the interval time since the last rabies vaccination increased (100% in the 2–3 years group, 42.9% in the >7 years group). The seroprotection rate was low in the group receiving PCECV-KMB.

#### 3.4.2. Three-Dose Sample

Among the 58 participants receiving three doses of the rabies vaccine, 50 were antibody-positive (86.2%). Table 5 shows the characteristics of the participants receiving three doses of the vaccine, which included 37 who received PVRV and 21 who received PCECV. One antibody-negative participant received PVRV (Verorab), and the seroprotection rate was low in the group receiving PCECV-KMB.

#### 3.4.3. Four-Dose Sample

Among the 17 participants receiving four doses of rabies vaccine, 16 were antibody-positive (94.1%) and 1 was antibody-negative. As shown in Table 6, the single negative participant, who was 66 years old at enrollment and 58 years old at primary vaccination, received four doses of PCECV-KMB (0, 1, 7 months, 4 years), and the interval time from the last dose was 3 years.

#### 3.4.4. Sample Receiving Five or More Doses

Among the 12 participants receiving five or more doses of rabies vaccine, all were antibody-positive (100%).

## 4. Discussion

PrEP is recommended for individuals who are frequently exposed to or at high risk for rabies virus and other lyssaviruses. These include laboratory personnel working with lyssaviruses, veterinarians, individuals in contact with wild animals such as bats, and to a lesser extent, those working or traveling in rabies-endemic areas [15,16,17].

This study provides compelling evidence for the long-term persistence of rabies virus neutralizing antibodies following PrEP among Japanese adult travelers. Notably, 86.6% of participants remained seropositive, with a median interval of 4 years since the last vaccination, indicating durable immune responses in most individuals. Participants who received three or more doses exhibited significantly higher geometric mean titers (GMTs) compared to those who received only two doses, suggesting a dose-dependent enhancement of immunogenicity [18,19].

Although the two-dose PrEP schedule has been globally accepted since its endorsement by the WHO and CDC in 2018, our data raise important questions about its long-term efficacy in certain subpopulations. Among individuals vaccinated with PCECV-KMB—formerly approved for subcutaneous administration in Japan—a notable proportion were found to be seronegative after a long post-vaccination interval. This suggests that both the vaccine formulation and the route of administration may influence immunogenicity. These findings align with those of previous studies indicating that intramuscular or intradermal administration may elicit more robust and sustained immune responses [20].

PCECV-KMB differs from internationally used rabies vaccines, such as Rabipur and Verorab, in several key aspects. It utilizes the Flury HEP strain (a highly attenuated, low-replication virus) and is produced in chick embryo cells. In contrast, Rabipur and Verorab both use the Flury LEP (a partially attenuated strain) but differ in their cell–substrates: Rabipur is also produced in chick embryo cells, while Verorab is produced in Vero cells. Additionally, PCECV-KMB was approved only for subcutaneous injection in Japan, with a three-dose schedule on day 0, day 28, and six months later, diverging from the WHO-recommended intramuscular regimen [21]. These differences in viral strain subtype, cell–substrate, administration route, and dosing schedule may contribute to the reduced immunogenicity and antibody persistence observed in PCECV-KMB recipients.

Recent literature supports the effectiveness of a two-dose rabies PrEP schedule for long-term immune priming [22,23,24], and several reports suggest that booster doses may not be necessary for individuals who complete the two-dose regimen [25,26,27,28,29]. While two-dose PrEP is generally effective, CDC guidelines recommend a booster for individuals at persistent high risk [30,31]. Our findings support this approach, particularly for those vaccinated with PCECV-KMB. Clinical decisions regarding additional PrEP doses should consider the time elapsed since vaccination, the availability of antibody testing, and the specifics of vaccine type and administration route. This consideration is especially important for travelers to rabies-endemic regions or individuals with limited access to PEP [32].

This study has several limitations. First, it was an observational study targeting individuals who had received PrEP more than two years ago, so baseline immune status before vaccination could not be confirmed. The absence of post-vaccination baseline antibody data limits our ability to differentiate between insufficient initial immunogenicity and waning immunity over time. Second, participants were recruited from travelers who visited the Travellers’ Medical Center at Tokyo Medical University Hospital, resulting in a relatively small sample size. This recruitment method may have introduced a “healthy vaccinee bias,” as individuals who proactively seek travel medicine services tend to have higher health literacy and fewer comorbidities, such as immunosuppressive conditions, compared to the general population. Furthermore, although the evaluated PrEP schedule involved three intramuscular doses of rabies vaccine, only 10 participants had received the two-dose regimen due to prior vaccination history. Finally, humoral immunity represents only one aspect of the immune response to rabies vaccination. The cellular immune response remains poorly understood and is difficult to assess. Therefore, in accordance with current recommendations, we considered rabies antibody measurement to be the most practical surrogate for evaluating post-vaccination immune protection [33].

## 5. Conclusions

This study confirms the long-term persistence of antibodies following rabies PrEP among Japanese travelers. Antibody levels were significantly influenced by the number of vaccine doses, with reduced persistence observed in individuals who received only two doses. When valid antibody titers are confirmed through testing, additional PrEP booster doses may not be necessary. However, in cases where individuals received only two doses, were vaccinated a long time ago, or were administered locally approved rabies vaccines such as PCECV-KMB, a risk-based assessment should be conducted. In such cases, consideration of booster doses may be warranted—particularly for those planning to travel to rabies-endemic regions.

## Figures and Tables

**Table 1:** Characteristics of the study participants (N = 97).

Characteristics	n (%)
Sex	
Male	53 (54.6)
Female	44 (45.4)
Age at primary vaccination (years)	Median age (IQR): 35 (29–44)
<50	85 (87.6)
≥50	12 (12.4)
Vaccination doses	
2	10 (10.3)
3	58 (59.8)
4	17 (17.5)
5 or more	12 (12.4)
Vaccine type	
PCECV-KMB	33 (34.0)
PCECV-Rabipur	12 (12.4)
PVRV	51 (52.6)
Unknown	1 (1.0)
Elapsed time from last vaccination (years)	Median time (IQR): 4 (3–7)
2–3	36 (37.1)
4–9	53 (54.6)
10–18	8 (8.3)

N, number of participants; IQR, interquartile range. Vaccine type: classification was based on the product used for the first dose of the vaccination series.

**Table 3:** ANOVA Results for LogAb Response.

Factor	Sum of Squares	df	F Value	Pr (>F)	Significance
Intercept	0.6848	1	2.3566	0.128	
Age Group	0.4398	1	1.5135	0.222	
Vaccination Dose Group	8.9440	3	10.2601	<0.001	***
Interval Group	1.6533	2	2.8449	0.064	.
Sex	0.0568	1	0.1954	0.660	
Vaccine Type	3.7207	3	4.2682	0.007	**
Residuals	24.9895	86			

Significance codes: *** *p* < 0.001, ** *p* < 0.01, . *p* < 0.1.

**Table 4:** Anti-rabies virus antibody (VNA) level in each participant receiving two-dose vaccination (n = 10).

Vaccine Name and Schedule (d)	Sex	Age at Primary Vaccination (y)	Elapsed Time (y)	VNA Level (IU/mL)
PVRV (0, 7 d)	M	32	2	4.00
PVRV (0, 7 d)	F	29	3	1.35
PVRV (0, 7 d)	M	39	3	0.62
Rabipur (0, 21 d)	F	33	7	0.88
PCECV-KMB (0, 28 d)	M	55	7	0.32 ^1^
PCECV-KMB (0, 28 d)	M	26	8	0.09 ^1^
PCECV-KMB (0, 28 d)	M	40	8	0.46 ^1^
PCECV-KMB (0, 28 d)	F	26	11	1.00
PCECV-KMB (0, 21 d)	F	27	16	0.92
PCECV-KMB (0, 21 d)	F	33	17	0.34 ^1^

^1^ VNA concentration was negative. VNA: viral neutralizing antibody; M: male; F: female; PVRV: purified Vero cell rabies vaccine (Verorab); PCECV: purified chick embryo cell vaccine (KM Biologics or Rabipur); d, day; y, year.

**Table 5:** Anti-rabies virus antibody (VNA) level in each participant receiving three-dose vaccination (n = 58).

Vaccine Name and Schedule (d/m/y)	Elapsed Time (y)	Seroprotection	VNA Level (IU/mL)		
			<0.5	0.5–0.99	≥1.0
PVRV (0, 7 d, 21–28 d)	2–10	36/37	1 ^1^	13	23
Rabipur (0, 7 d, 21–28 d)	2–6	5/5	0	2	3
PCECV-KMB (0, 7 d, 21–28 d)	4–9	4/7	3 ^1^	1	3
PCECV-KMB (0, 1 m, 6–12 m)	2–9	4/8	4 ^1^	1	3
PCECV-KMB (0, 4 y, 6 y)	18	1/1	0	0	1

^1^ VNA concentration was negative. PVRV: purified Vero cell rabies vaccine (Verorab, Abhayrab); PCECV: purified chick embryo cell vaccine (Rabipur, KM Biologics); d, day; m, month; y, year.

**Table 6:** Anti-rabies virus antibody (VNA) level in each participant receiving four-dose vaccination.

Vaccine Name and Schedule (d/m/y)	Sex	Age at Primary Vaccination (y)	Elapsed Time (y)	VNA Level (IU/mL)
PVRV (0, 7 d, 28 d, 2 y)	M	50	2	133.96
PVRV (0, 7 d, 28 d, 4 y)	M	55	2	32.00
PVRV (0, 7 d, 28 d, 7 y)	M	21	2	1.54
PVRV (0, 7 d, 28 d, 6 y)	M	49	4	3.22
PVRV (0, 7 d, 28 d, 1 y)	F	40	5	2.48
PVRV (0, 7 d, 28 d, 1 y)	M	55	5	2.28
PVRV (0, 14 d, 28 d, 8 m)	F	38	8	0.57
PVRV (0, 14 d, 28 d) Rabipur (1 y)	M	41	4	53.85
Rabipur (0, 7 d, 28 d, 1 y)	M	47	4	0.71
Rabipur (0, 7 d, 28 d, 1 y)	M	36	5	108.49
Rabipur (0, 7 d, 28 d, 3 y)	M	50	9	2.00
Rabipur (0, 7 d, 28 d) Chirorab (1 y)	M	29	2	400
PCECV-KMB (0, 1 m, 6 m, 3 y)	F	48	2	2.71
PCECV-KMB (0, 7 d, 8 m, 9 y)	M	46	2	1.00
PCECV-KMB (0, 1 m, 7 m, 4 y)	F	58	3	0.30 ^1^
PCECV-KMB (0, 1 m, 9 m) Pasteur (7 y)	F	30	12	15.32
Unknown (0, 1 m, 10 m) PCECV-KMB (7 y)	F	34	8	1.76

^1^ VNA concentration is negative. PVRV: purified Vero cell rabies vaccine (Verorab, Abhayrab); PCECV: purified chick embryo cell vaccine (Rabipur, KM Biologics); M, male; F, female; d, day; m, month; y, year.

## Data Availability

Data supporting the study are available from the corresponding author upon request.

## Abbreviations

The following abbreviations are used in this manuscript:

PrEP	pre-exposure prophylaxis
PEP	post-exposure prophylaxis
PVRV	purified Vero cell rabies vaccine
PCECV	purified chick embryo cell rabies vaccine
WHO	World Health Organization
GMT	geometric mean titer
CI	confidence interval
IQR	interquartile range
N	number of participants
VNA	viral neutralizing antibody
M	male
F	female
d	day
m	month
y	year
