# Correction: Fukushima et al. Long-Term Immunogenicity of Rabies Pre-Exposure Prophylaxis in Japanese Adult Travelers: Comparison of Dosing Regimens. *Vaccines* 2025, *13*, 1169

**DOI:** 10.3390/vaccines14060472

**Published:** 2026-05-26

**Authors:** Shinji Fukushima, Akira Nishizono, Takehiro Hashimoto, Atsuo Hamada

**Affiliations:** 1 Travellers’ Medical Center, Tokyo Medical University Hospital, 6-7-1 Nishi-shinjuku, Shinjuku-ku, Tokyo 160-0023, Japan; 2 Department of Microbiology, Faculty of Medicine, Oita University, 1-1 Idaigaoka, Hasama-machi, Yufu, Oita 879-5593, Japan; 3 Research Center for Global and Local Infectious Diseases, Oita University Hospital, 1-1 Idaigaoka, Hasama-machi, Yufu, Oita 879-5593, Japan; 4 Hospital Infection Control Center, Oita University Hospital, 1-1 Idaigaoka, Hasama-machi, Yufu, Oita 879-5593, Japan

The authors would like to make the following corrections to this published paper [1].

In the original publication, there was a mistake in Table 1 as published. “Median time” for “Elapsed time from last vaccination (years)” was incorrectly presented as “Median age”, and one of the values (10–18) for “Elapsed time from last vaccination (years)” was incorrectly presented as (10–15). The corrected Table 1 appears below.

Based on the corrections to Table 1, corresponding changes are required to the Abstract and Discussion. Both sections incorrectly stated that the median interval since vaccination was 8.5 years. The correct value is “a median interval of 4 years since the last vaccination”. A correction has been made to the Abstract:

“Among 97 participants, 86.6% remained seropositive, with a median interval of 4 years since the last vaccination.”

A correction has also been made to the Discussion (Paragraph 2):

“Notably, 86.6% of participants remained seropositive, with a median interval of 4 years since the last vaccination, indicating durable immune responses in most individuals.”

There was a mistake in Table 2 as published. The seroprotection rates for the different vaccines were incorrectly presented. The corrected Table 2 appears below.

There was a mistake in Table 5 as published. The VNA levels were incorrectly categorized and were incorrectly presented for PVRV. The corrected Table 5 appears below.

The sentence in the Results, Section 3.2, Paragraph 1 reporting antibody levels as 24 (24.7%), 34 (34.4%), 9 (9.3%), and 18 (18.6%) is incorrect. The sentence should be as follows:

“Of these, 24 (28.6%), 33 (39.3%), 9 (10.7%), and 18 (21.4%) had antibody levels of 0.5–0.99, 1.0–2.49, 2.5–3.99, and ≥4.0 IU/mL, respectively.”

The authors state that the scientific conclusions are unaffected. These corrections were approved by the Academic Editor. The original publication has also been updated.

## Figures and Tables

**Table 1:** Characteristics of the study participants (N = 97).

Characteristics	n (%)
Sex	
Male	53 (54.6)
Female	44 (45.4)
Age at primary vaccination (years)	Median age (IQR): 35 (29–44)
<50	85 (87.6)
≥50	12 (12.4)
Vaccination doses	
2	10 (10.3)
3	58 (59.8)
4	17 (17.5)
5 or more	12 (12.4)
Vaccine type	
PCECV-KMB	33 (34.0)
PCECV-Rabipur	12 (12.4)
PVRV	51 (52.6)
Unknown	1 (1.0)
Elapsed time from last vaccination (years)	Median time (IQR): 4 (3–7)
2–3	36 (37.1)
4–9	53 (54.6)
10–18	8 (8.3)

N, number of participants; IQR, interquartile range. Vaccine type: classification was based on the product used for the first dose of the vaccination series.

**Table 2:** Seroprotection rate and geometric mean antibody level (GMT).

Characteristics	Seroprotection		GMT (95%CI)	
	n/N	% (95%CI)		
Total	84/97	86.6 (78.2–92.7)	1.65	(1.22–2.23)
Sex				
Male	46/53	86.8 (74.7–94.5)	1.81	(1.10–2.98)
Female	38/44	86.4 (72.6–94.8)	1.47	(1.09–1.99)
	*p* = 1.000		*p* = 0.951	
Age at primary vaccination (years)				
<50	75/85	88.2 (79.4–94.2)	1.67	(1.24–2.25)
≥50	9/12	75.0 (42.8–94.5)	1.52	(0.39–5.92)
	*p* = 0.201		*p* = 0.558	
Vaccination doses				
2	6/10	60.0 (26.2–87.8)	0.64	(0.31–1.33)
3	50/58	86.2 (74.6–93.9)	1.15	(0.88–1.51)
4	16/17	94.1 (71.3–99.9)	5.58	(1.85–16.8)
5 or more	12/12	100 (73.5–100)	3.64	(1.60–8.28)
	*p* < 0.05		*p* < 0.05	
Vaccine type				
PCECV-KMB	21/33	63.6 (45.1–79.6)	0.93	(0.57–1.51)
Rabipur	12/12	100 (73.5–100)	3.96	(0.92–17.1)
PVRV	50/51	98.0 (89.6–100)	1.95	(1.40–2.72)
Unknown	1/1		1.75	
	*p* < 0.05		*p* = 0.159	
Elapsed time from last vaccination (years)				
2–3	32/36	88.9 (73.9–96.9)	2.23	(1.25–3.97)
4–9	45/53	84.9 (72.4–93.3)	1.30	(0.90–1.88)
10–18	7/8	87.5 (47.3–99.7)	2.05	(0.78–5.41)
	*p* = 0.900		*p* = 0.444	

Fisher’s exact test was used to compare antibody prevalence rates between two groups, and the Holm test and Cochran–Armitage test were used to compare antibody prevalence rates between multiple groups. Comparisons of GMT between two groups were performed using the Wilcoxon rank sum test, and multi-group comparisons of GMT were performed using the Kruskal–Wallis test. Vaccine type: classification was based on the product used for the first dose of the vaccination series.

**Table 5:** Anti-rabies virus antibody (VNA) level in each participant receiving three-dose vaccination (n = 58).

Vaccine Name and Schedule (d/m/y)	Elapsed Time (y)	Seroprotection	VNA Level (IU/mL)		
			<0.5	0.5–0.99	≥1.0
PVRV (0, 7 d, 21–28 d)	2–10	36/37	1 ^1^	13	23
Rabipur (0, 7 d, 21–28 d)	2–6	5/5	0	2	3
PCECV-KMB (0, 7 d, 21–28 d)	4–9	4/7	3 ^1^	1	3
PCECV-KMB (0, 1 m, 6–12 m)	2–9	4/8	4 ^1^	1	3
PCECV-KMB (0, 4 y, 6 y)	18	1/1	0	0	1

^1^ VNA concentration was negative. PVRV: purified Vero cell rabies vaccine (Verorab, Abhayrab); PCECV: purified chick embryo cell vaccine (Rabipur, KM Biologics); d, day; m, month; y, year.
